# Cost of Myopia Correction: A Systematic Review

**DOI:** 10.3389/fmed.2021.718724

**Published:** 2021-12-03

**Authors:** Li Lian Foo, Carla Lanca, Chee Wai Wong, Daniel Ting, Ecosse Lamoureux, Seang-Mei Saw, Marcus Ang

**Affiliations:** ^1^Singapore National Eye Centre, Singapore, Singapore; ^2^Singapore Eye Research Institute, Singapore, Singapore; ^3^Duke–NUS Medical School, National University of Singapore, Singapore, Singapore; ^4^Escola Superior de Tecnologia da Saúde de Lisboa (ESTeSL), Instituto Politécnico de Lisboa, Lisboa, Portugal; ^5^Comprehensive Health Research Center (CHRC), Escola Nacional de Saúde Pública, Universidade Nova de Lisboa, Lisboa, Portugal; ^6^NUS Saw Swee Hock School of Public Health, Singapore, Singapore

**Keywords:** myopia, costs, spectacles, contact lenses, refractive surgeries declaration

## Abstract

Myopia is one of the leading causes of visual impairment globally. Despite increasing prevalence and incidence, the associated cost of treatment remains unclear. Health care spending is a major concern in many countries and understanding the cost of myopia correction is the first step eluding to the overall cost of myopia treatment. As cost of treatment will reduce the burden of cost of illness, this will aid in future cost-benefit analysis and the allocation of healthcare resources, including considerations in integrating eye care (refractive correction with spectacles) into universal health coverage (UHC). We performed a systematic review to determine the economic costs of myopia correction. However, there were few studies for direct comparison. Costs related to myopia correction were mainly direct with few indirect costs. Annual prevalence-based direct costs for myopia ranged from $14-26 (USA), $56 (Iran) and $199 (Singapore) per capita, respectively (population: 274.63 million, 75.15 million and 3.79 million, respectively). Annually, the direct costs of contact lens were $198.30-$378.10 while spectacles and refractive surgeries were $342.50 and $19.10, respectively. This review provides an insight to the cost of myopia correction. Myopia costs are high from nation-wide perspectives because of the high prevalence of myopia, with contact lenses being the more expensive option. Without further interventions, the burden of illness of myopia will increase substantially with the projected increase in prevalence worldwide. Future studies will be necessary to generate more homogenous cost data and provide a complete picture of the global economic cost of myopia.

## Introduction

Myopia is one of the leading causes of visual impairment in the world (1, 2). The prevalence of myopia ranges from 15 to 49% in adult populations, and ranges from 20 to 90% in children, adolescents and young adults (3–7). Studies estimate that myopia will affect 50% (4.7 billion) of the world's population by 2050, with 10% (1 billion) having high myopia (≤ -5.00 Dioptres) (8–10) Correction of myopia with spectacles, contact lenses and refractive surgeries therefore play an increasingly important role in society, as uncorrected myopia results in reduction of visual acuity leading to impaired visual functioning (11).

However, there are significant costs associated with optical correction, treatment to retard myopia progression and treatment of myopia related complications, including pathologic myopia, cataract, glaucoma and retinal detachment (12–16). With increasing demand for the limited healthcare resources globally, an understanding of the economic cost associated with the treatment of myopia is important for further cost-benefit analysis and policy making decisions. This will aid and justify in the allocation of invaluable healthcare resources to the treatment of myopia, in order to reduce the economic burden of this illness.

We aim to perform an evidence-based review of the economic costs associated with the correction of myopia.

## Sources and Methods of Literature Search

**Figure 1:**
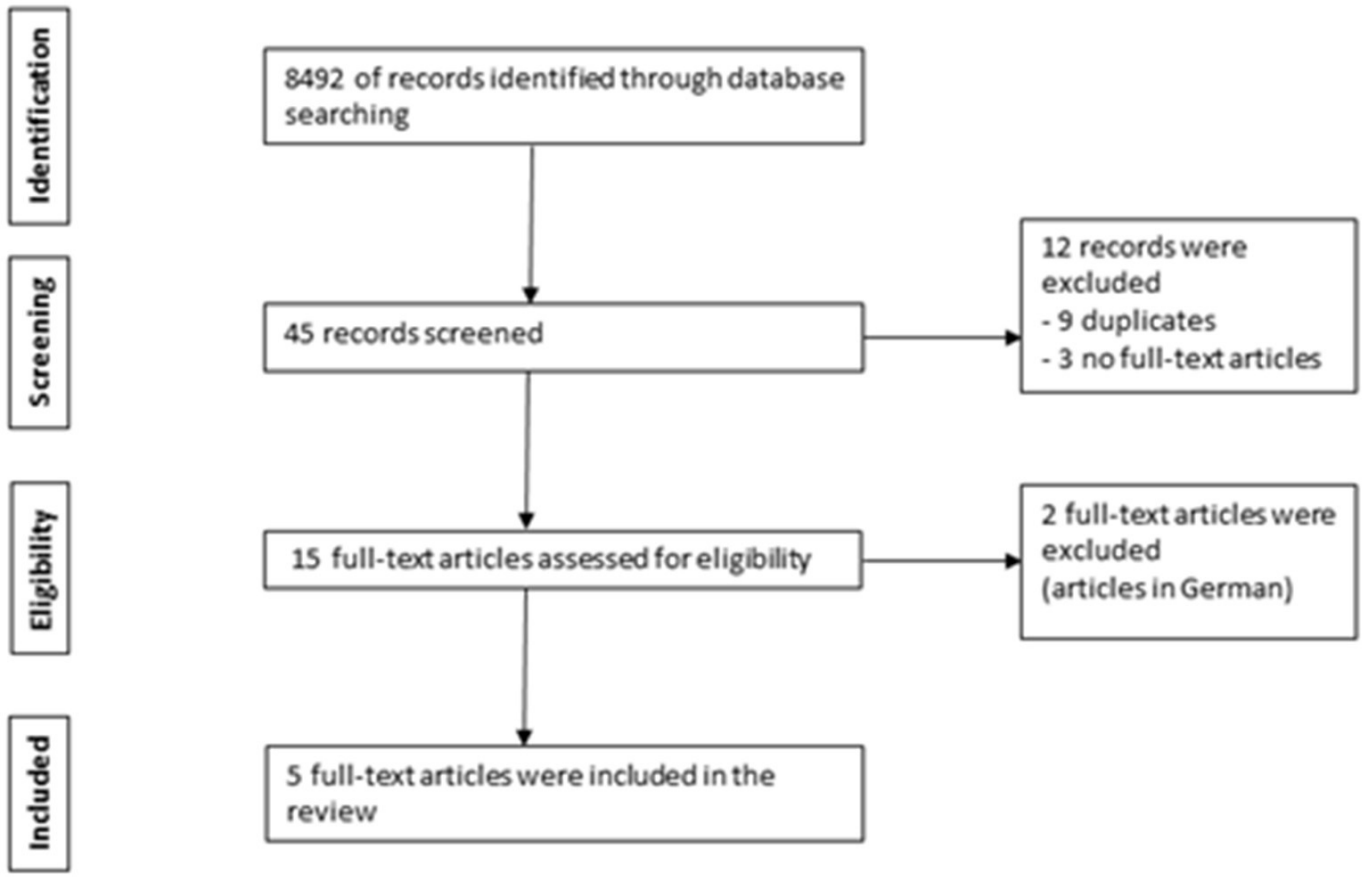
Flowchart of the review article selection process.

We conducted a systematic review of relevant literature articles in accordance with the Preferred Reporting Items for Systematic Reviews and Meta-analysis (PRISMA) guidelines (17). Several electronic databases (PubMed, ScienceDirect, Cochrane Library, and Web of Science databases) were searched to identify English language articles up to 29 February 2020 on costs associated with myopia correction treatment. The search used the keywords “myopia,” “short-sightedness” or “near-sightedness” combined with “cost” or “economic burden.” Original full-text articles in English were included if costs were quantified in relation to myopia correction, including: myopia correction (spectacles, contact lenses, refractive surgeries). 8,492 titles were retrieved through database searching. Forty five relevant records were reviewed with 12 records excluded (9 duplicates and 3 with no full-text available). Fifteen full-text articles were assessed for eligibility with 2 non-English articles excluded (articles in German). Articles that did not fulfill the inclusion criteria were excluded. Five eligible full-text articles were included in this review (18–22). The review article selection process is illustrated as a flowchart in Figure 1. The Asian studies comprised of 2 from Singapore while the non-Asian studies comprised of one from each of the following countries: United States of America (USA), Iran and Spain.

A 20-items Consensus Health Economic Criteria (CHEC)-extended checklist was used to evaluate the overall quality of included studies (23, 24). Scoring was performed by assigning a score of 1 (yes), 0 (no), 2 (not applicable) to each item and the total scores were summed to generate the overall quality score (0–100%). The total quality score for each study was categorized into low, moderate, good and excellent with cut-off value of <50, 51–75, 76–95 and >95, respectively. Only moderate, good and excellent quality studies were included as higher scores denote lower risk of bias. Two independent reviewers conducted the assessment (LLF and CL) and the interrater-agreement was evaluated using κ from STATA/IC 11.1 (25). The interpretation of the κ was based on a scale which indicates poor, slight, fair, moderate, substantial and perfect agreement with κ levels <0.0, 0.0–0.20, 0.21–0.40, 0.41–0.60, 0.61–0.80 and ≥0.81, respectively (26). Of the included studies, 4 were good in quality (76.5–95) and 1 was excellent (100). The interrater-reliability κ was moderate in 1 study (0.44), substantial in 2 studies (0.63, 0.64) and perfect in 2 studies (1, 1).

Examples of costs assessed included optical correction devices/procedures (spectacles, contact lenses, refractive surgeries), visits to professional services (transportation and fees) and time spent and loss of productivity while seeking treatment.

All costs are quoted in US dollars ($). Conversion rate used was Euro to USD = 1:1.12 (22, 27) and Pound sterling to USD = 1:1.31 (28), using average 2019 exchange rates (29).

## Results

The costs for myopia correction are shown in Table 1.

**Table 1:** Summary table of reviewed articles (treatment of Myopia-Myopia correction: *n* = 5).

**Treatment of Myopia (Myopia correction)**											
**No**	**References**	**Year**	**Country**	**Type of study**	**Costs**	**Sample size (** ***n*** **)**	**Age (Years)**	**Method of ascertaining cost**	**Prevalence of Myopia (%)**	**Direct cost ($)**	**Indirect cost ($)**
1	Ruiz-Moreno and Roura (27)	2009	Singapore	Cross-sectional study	Myopia correction	301	12–17	Parent and Self questionnaire	NA	Annual direct cost Mean (Per patient) = $147.8 ± 209.1 (CI, $124.3–172.1) Median (Per patient) = $83.3 Mean cost per pair of spectacles $82.1 ± 40.8 (CI, $77.8–86.5) Mean annual cost of contact lenses $378.1 ± 377.1 (CI, $281.4–474.6).	NA
2	Zheng YF et al. (30)	2013	Singapore	Cross-sectional study	Myopia correction	113	52.6 ± 7.8	Questionnaire	Age 0-4 = 10% 5–9 = 30% 10–14 = 60% 15–24 = 80% 25–39 = 90% 40–49 = 45% 50–59 = 35% 60–80+=30%	Annual direct cost Mean (Per patient) = $709 Annual direct cost Singapore = $755 million Urban Asia = $328 billion Lifetime per capita cost (disease of 0–80 years) $232–17,020	
3	Vitale S et al. (31)	2006	USA	Cross-sectional	Myopia correction	13211	≥12	NHANES and fees schedule and expenditure data	NA	Annual direct cost All = $3.9–7.2 billion Persons age > 65 = $780 million	NA
4	Morgan et al. (7)	2002	Spain	Cross-sectional	Myopia correction	40 (80% Myopia)	Mean 32	Questionnaire markov model	NA	Total direct cost* (10, 20 and 30 years) LASIK = $ 3341.96; 3368.75; 3385.71 Spectacles = $ 1091.07; 1623.21; 1960.71 Contact lens = $ 3019.64; 4723.21; 5779.46	NA
5	Malec et al. (32)	2018	Iran	Cross-sectional	Myopia correction	120 (60.83% Myopia)	≥23	Interview	Age <14 = 3.6% 15–19 = 16.5% 20–29 = 22.0% >60 = 32.8%	Total annual direct cost* Spectacles = $342.5 ± 8.41 Contact lenses = $198.30 ± 0.12 Refractive surgery = $19.10 ± 1.2 Lifetime direct cost* Spectacles = $9373.5 ± 230.1 Contact lenses = $5203.10 ± 256.3 Refractive surgery = $568.1 ± 64.6 Annual direct cost* Mean (Per patient) = $309 Annual direct cost All ages = $4.2 billion Persons age <14 = $196 million Persons age 15-19 = $337 million Persons age 20-29 = $3043 million Persons age > 60 = $628.55 million	Total annual indirect cost* Spectacles = $12112.10 Contact lenses = $3045.20 Refractive surgery = $113.60 Lifetime indirect cost* Spectacles = $331380.60 Contact lenses = $79762.20 Refractive surgery = $2789.10
										Annual and lifetime total costs* (direct and indirect) Spectacles = $12454.6; 340754.10, Contact lenses = $3243.5; 84965.30 Refractive surgery = $132.7; 3357.20	

* *Include all types of refractive error (myopia, hyperopia and astigmatism)*.

The average direct costs of myopia correction in Singapore children aged 12–17 years from the SCORM study (Singapore Cohort study of the Risk factors of Myopia) were $147.80 per year per myopia patient, $82.10 per pair of spectacles and $378.10 per year for contact lenses (18).

In Singapore adults aged ≥40 years, the mean direct cost of myopia correction was $709 per year per patient. This estimate translates into an annual economic burden of $755 million in Singapore. Refractive correction, comprising of optometry visits, spectacles and contact lenses, were the most significant, accounting for 65.2% of the total costs (19). The remaining costs comprise of refractive surgeries and complications related to it as well as contact lens use.

In USA, the annual direct country-wide cost of correcting distance vision impairment was estimated to be between $3.9 and $7.2 billion, with $780 million per annum for persons >age 65 years (33). The National Health and Nutrition Examination Survey (NHANES) was an ongoing, nationally representative survey of 14,203 participants aged ≥ 12 years (32, 33). The cost calculations were based on single-vision spectacles, without including other refractive correction options. Hence, this cost would be much higher if contact lenses and refractive surgeries were taken into account. As the annual costs from the earlier Singapore study were based on all forms of corrections, direct comparison is inequitable. In addition, due to the study's methodology for distant vision correction, subjects with pure astigmatism without myopia were also included in the cost calculations.

In two other studies (21, 22), the costs of refractive correction were computed by including other refractive errors (hyperopia and astigmatism). While the costs of each modality for myopia correction alone could not be determined, they provide insights to the general cost for refractive correction in the country.

In a Spanish study, the direct cost of spectacles, contact lenses and LASIK were evaluated (22). It was reported that the total direct (medical and non-medical) cost over 10, 20, and 30 years (5% discount rate) for contact lens was $3019.64; 4723.21; 5779.46, LASIK was $3341.96; 3368.75; 3385.71 and spectacles was $1091.07; 1623.21; 1960.71 (22). This was a small study of 40 subjects from one city in Alicante, with 80% myopes (12.5% hyperopes and 42.5% astigmatic). This study was conducted in 2002 and hence costs might not be representative of the current market, particularly the cost of cleaning and fitting contact lens and transport system with technological advancements.

In a recent Iranian study, 120 subjects aged ≥ 23 years were interviewed in a hospital and the lifetime direct costs of spectacles, contact lenses and refractive surgeries were $9373.50, $5203.10, and $568.10, respectively (21). The annual direct costs of refractive correction per patient and for each of the three modalities were $309, $342.50, $198.30, and $19.10, respectively. Annually, direct cost of myopia correction was estimated to be $4.2 billion in Iran. Indirect costs in this study were estimated using the human capital approach, by ascertaining lost productivity due to the complication, maintenance, repair and travel costs as a measure of patient's and caregiver's lost earnings (34). Annually, the indirect costs were $12112.10, $3045.20, and $113.60, respectively with the main bulk contributed by patient's and caregiver's opportunity cost. However, it was not clear from the study regarding the basis and role of caregiver's costs calculation in optical correction and no justification was offered for the high indirect costs from spectacles, considering it is least prone to complications. In addition, cost calculations for each refractive correction modality were generalized to all forms of refractive errors, it was challenging to estimate the cost generated from myopia only.

**Figure 2:**
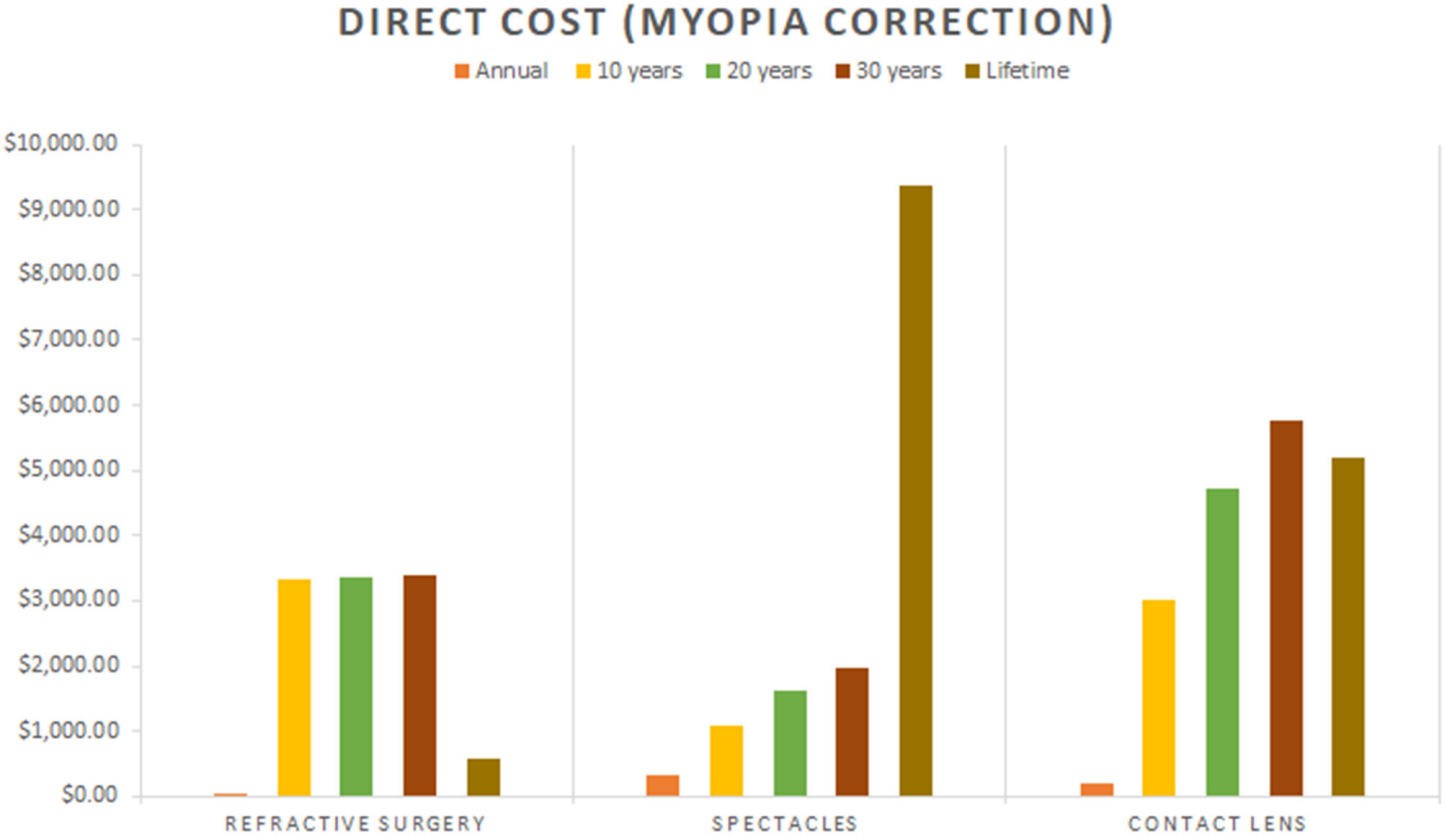
Cost of myopia correction modalities (Refractive surgeries, Spectacles, Contact lens).

Out of the three groups of myopia correction modalities reported in the studies (18, 21, 22), contact lens and spectacles appeared to be generally more costly than refractive surgeries (Figure 2). Annually, the direct costs of contact lens and spectacles were $198.30-$378.10 and $342.50, respectively while refractive surgeries was $19.10 (18, 21).

In Singapore, while the annual direct cost of myopia correction to the individual is the lowest compared to diabetic retinopathy and wet age-related macular degeneration (AMD) (18, 19, 35, 36), the nation's annual direct cost of myopia correction ($755 million) alone far exceeded other ocular diseases including acute primary angle closure glaucoma ($0.26–0.29 million), dry eyes ($1.51–1.52 million) and wet AMD ($96.8–120.7 million) (Table 2) (18, 19, 35, 36, 41, 42).

**Table 2:** Cost of ocular diseases in Singapore.

**Eye diseases in Singapore**	**Annual direct cost in Singapore ($)**	**Mean annual direct cost per patient ($)**
Diabetic retinopathy (37)	NA	$863.65–2660.15
Acute primary angle closure glaucoma (38)	$0.26–0.29 million	NA
Dry eyes (39)	$1.51–1.52 million*	NA
Wet AMD (40)	$96.8–120.7 million	$6902.20
Myopia correction (27, 30)	$755 million	$147.80–709

* *Singapore National Eye Centre only*.

## Discussion

In this review, we found 5 studies addressing the cost of myopia correction (18, 19, 21, 22, 33), which are generally direct costs from spectacles, contact lens and refractive surgeries. The per capita annual cost of myopia correction was low in USA, moderate in Iran and high in Singapore. Indirect costs in myopia correction are mainly related to complications, particularly with contact lens use, including cost of treatment, loss of productivity secondary to complications and its associated travel costs (21). We found that the annual direct costs of myopia correction in USA, Iran and Singapore were substantial at $3.9–7.2 billion, $4.2 billion and $755 million, respectively. This translated to $14–26 (USA), $56 (Iran) and $199 (Singapore) per capita, respectively (population: 274.63 million, 75.15 million and 3.79 million, respectively) (19, 21, 43). Most costs related to myopia correction were direct costs, with contact lens appearing to be generally more costly compared to other modalities.

We found few studies to adequately address this topic and limited studies using similar costs definitions for comparison. Firstly, there was a limited representation of studies globally, with 2 from Asia (Singapore) and 3 from Europe (Spain), USA and Middle East (Iran), respectively. Secondly, different methodologies and cost definitions were used for cost calculations and many studies did not assessed indirect costs in detail.

The World Health Organization (WHO) considers spectacles or contact lenses as functioning interventions (44), with spectacles being also considered as an assistive device which is part of the WHO Priority Assistive Products List (45). As health care spending is a major concern in many countries, understanding the cost of myopia correction is the first step eluding to the overall cost of myopia treatment. Moreover, among the worldwide population with moderate or severe vision impairment, uncorrected refractive error was the highest at 116.3 to 123.7 million (46, 47), with the cost of coverage gap for unaddressed refractive error and cataract estimated to be $14.3 billion globally (45). As cost of treatment will reduce the burden of cost of illness, this will aid in future cost-benefit analysis and the allocation of healthcare resources, including considerations in integrating eye care (refractive correction with spectacles) into universal health coverage (UHC) (45). This is particularly important in Asian developing countries where there is high prevalence of myopia with low accessibility to spectacles.

Although the cost of myopia to an individual may not be very high, the cost of myopia to the nation is one of the highest as the prevalence of myopia is higher than many other diseases. The high prevalence of myopia plays an important role in determining the economic cost of the treatment of myopia in each country. In East and Southeast Asia, the prevalence of myopia was reported to be as high as 80–90% in adolescents of age of 17–18 (7). In contrast, 20–40% was reported in developed western countries (7, 20, 40, 48–50). Hence while the magnitude of direct cost of refractive correction was greater in USA and Iran than in Singapore, the per capita cost was lesser at $14–26 and $56 vs. $199 (19, 31).

Other factors that could account for variation in costs include country-specific costs, different methodologies, study subject's characteristics (including age), timeline, varying costs of living and socioeconomic status. However, due to limited studies available, it would be challenging to explore the influence of these factors. As the governments in most countries are unlikely to be able to monitor spectacle or contact lens sales, future cost data can be obtained by considering cross-sectional rapid assessment protocols, targeting for instance high schools.

In Singapore, although the annual direct cost of myopia correction to the individual is lowest amongst diabetic retinopathy and wet AMD (18, 19, 35, 36), the nation's annual direct cost of myopia correction alone far exceeded other ocular diseases including acute primary angle closure glaucoma, dry eyes and AMD (18, 19, 35, 36, 41, 42). This finding is not surprising and is attributed to the high prevalence of myopia in the country, with myopia expected to remain as the most common ocular condition with 2.393 million cases in 2040 (51).

Out of the three groups of myopia correction modalities, contact lens and spectacles seemed to be generally more costly than refractive surgery (18, 21, 22), with the exception of 1 study which did not justify the inclusion of high patient and caregiver opportunity costs from spectacles use (21). This is excluding the indirect costs of contact lens related complications (e.g., infective keratitis), including cost of treatment, loss of productivity secondary to complications and its associated travel costs. However, this cost is expected to be dynamic in view of technological advancement, economic forces, occupational and recreational requirements, individuals paying premium for factors such as aesthetics and quality as well as free or subsidized refractive correction by the government.

Contact lenses were mainly prescribed for the correction of myopia, with proportion as high as 94% (52). The three key cost components of contact lens wear are the professional fees, the cost of lenses and the cost of lens care solutions (38, 39). Spherical lenses have the lowest overall cost, followed by toric and multifocal lenses (39), with the true cost of lens wear (cost-per-wear) dependent on the frequency of use (38, 39). Generally, daily replacement contact lenses are more cost-effective on a part-time usage, while reusable lenses are more cost-effective on a full-time usage (38). With contact lens gaining popularity among the teenagers and young adults (52), together with the high prevalence of myopia in this age-group (3–7), the nation-wide costs of contact lenses are expected to rise in the near future.

We have reviewed the costs of optical correction of myopia. However, since the cost and burden to the nation is high, treatments to slow myopia progression and measures to prevent myopia and high myopia (including outdoor programs) are important to reduce the prevalence of myopia and subsequent costs of illness, including burden related to its complications.

Atropine eyedrops have shown strong evidence in myopia control while Orthokeratology, myopic defocus multizone contact lenses and spectacles have shown some effect (30, 37, 53–57). However, there is currently no literature reporting the treatment costs generated from Atropine use in children (53, 54). The use of myopia control treatment modalities will inevitably incur costs including equipment, professional services and the management of complications, particularly infective keratitis with contact lens use. Further studies, including cost-effectiveness randomized control trials of treatments for myopia progression will be necessary to evaluate this knowledge deficit.

## Limitations

For myopia correction, differentiating costs of optometry visits and refractive correction devices was difficult due to difference in studie's methodology. Another limitation includes the presence recall and non-response bias from retrospective design studies and the use of questionnaires/interviews. In addition, cost data reported in older studies may not be a reliable reflection of today's costs, due to various economic factors. Details of indirect costs were lacking. There were few studies available in the literature with limited representation globally.

## Further Studies

Future studies will be necessary to generate a more homogenous cost data and provide a more complete picture of the global economic cost of myopia treatment. These include cost of illness analysis, programmatic costs of spectacles correction in rural areas by non-governmental organizations and cost-effectiveness randomized control trials of treatments for myopia progression.

## Conclusion

Our systematic review provides insight on the costs of myopia correction. Annual prevalence-based direct costs for myopia correction are substantial, ranging from US$14–26 (USA), $56 (Iran) to $199 (Singapore) per capita. In Singapore, the annual direct cost of myopia correction alone far exceeded the costs of other ocular diseases including acute primary angle closure glaucoma, dry eyes and wet AMD due to high prevalence of disease. Without further interventions, the economic burden of illness of myopia will increase substantially with the projected increase in prevalence worldwide. Hence, myopia control treatment in children and measures to prevent myopia and high myopia will play an increasingly important role to reduce prevalence and costs of illness. Future studies will be necessary to generate a more homogenous cost data and provide a complete picture of the global economic cost of myopia.

## Data Availability Statement

The original contributions presented in the study are included in the article/Supplementary Material, further inquiries can be directed to the corresponding authors.

## Author Contributions

LF, CL, and S-MS: conception and design of study. LF, CL, CW, DT, EL, and MA: analysis and/or interpretation of data. LF, CL, S-MS, and MA: drafting the manuscript. CW, DT, EL, S-MS, and MA: revising the manuscript critically for important intellectual content. All authors contributed to the article and approved the submitted version.

## Conflict of Interest

The authors declare that the research was conducted in the absence of any commercial or financial relationships that could be construed as a potential conflict of interest.

## Publisher's Note

All claims expressed in this article are solely those of the authors and do not necessarily represent those of their affiliated organizations, or those of the publisher, the editors and the reviewers. Any product that may be evaluated in this article, or claim that may be made by its manufacturer, is not guaranteed or endorsed by the publisher.
